# From a "Resident's" Point of View

**Published:** 1903-03-14

**Authors:** 


### From a "Resident's" Point of View.

"Resident" writes under date 6th inst.:—You are perfectly right in your view that special provision is required for "drunk or dying," "drunk, damaged, and noisy" cases, and "Night Sister's" clever word-pictures should serve to convince even outsiders, among whom we shall soon have to count Mr. Sydney Holland if he continues to make such mistakes as he has done lately about things one would expect him to know quite well. Note, too, his remarkable question at the Hospitals Association meeting the other night: "What on earth could a medical student learn at a Poor-law infirmary?"

The point you are pressing appeals, no doubt, most acutely to patients and sisters, but every "Resident" of much experience must remember times when he has been sorely perplexed as to whether he should let a doubtful case go to the police station or admit it to an otherwise restful ward, or to a jealously guarded bed, which will thus be occupied, probably needlessly, for several days. A "drunk and slightly damaged" case is not so easy to get rid of once it has been admitted and learnt what a comfortable place a hospital bed may be.

What every hospital of any size ought to have is two or three rooms reserved absolutely for cases of this description, in which they can be simply "detained" until it is certain they are merely "drunks," or until, if obviously more or less injured as well, they have ceased to be noisy. If it is determined they are merely drunk, they can be turned out without further ceremony or trouble, while if the injuries appear to require in-patient treatment they can be formally admitted in due course.

The present system, or want of one, is not fair to patients, nurses, or residents, and if you can get it altered you will deserve their thanks, as well as remove a serious source of risk to the reputation of hospitals.

